# Deep learning for automated dental plaque index assessment: validation against expert evaluations

**DOI:** 10.1186/s12903-025-06350-2

**Published:** 2025-07-02

**Authors:** Jin-Sun Jeong, Kyeong-Seop Kim, Yu Gu, Da-Hyun Yoon, Meng Zhang, Ling Wang, Jeong-Hwan Kim

**Affiliations:** 1 School of Nursing and Rehabilitation, Cheeloo College of Medicine, Shandong University, Jinan, Shandong 250012 China; 2 Department of Biomedical Engineering, College of Science and Technology, Konkuk University, Chungju, Republic of Korea; 3 Department of Endodontics, School and Hospital of Stomatology, Cheeloo College of Medicine, Shandong University, Jinan, Shandong 250012 China; 4 Dental Hygienist, Seoul Deep Sleep Dental Clinic, Seoul, Republic of Korea; 5 Research and Development (R&D), Medicalpark. Inc, Yongin, Gyeonggi-do Republic of Korea; 6 U Tower 623, 767, Sinsu-ro, Suji-gu, Yongin-si, Gyeonggi-do Republic of Korea

**Keywords:** Oral hygiene, Dental plaque, Plaque index, Dental image, Deep learning, Artificial intelligence (AI)

## Abstract

**Background:**

The integration of artificial intelligence (AI) into healthcare has led to promising advancements in clinical decision-making and diagnostic accuracy. In dentistry, automated methods to evaluate oral hygiene measures, such as dental plaque detection, could improve patient care and streamline remote assessments.

**Objective:**

This study aimed to develop and evaluate a deep learning (DL)-based system that automatically detects and quantifies dental plaque, using a standardized plaque index, from intraoral images.

**Methods:**

Seventy participants were assessed using the Quigley-Hein plaque index, a clinical measure of plaque accumulation, following the application of a plaque-disclosing agent. Images were captured before and after dye application. Each tooth was labeled using the LabelMe software, indicating both tooth number and plaque presence for training and validation of the DL model. The performance of the DL-based system was statistically compared to the assessments of a highly experienced dentist (10 years) and a dental hygienist (1 year).

**Results:**

After data augmentation, the DL model achieved a micro-average accuracy of 73.67% and a macro-average accuracy of 65.15%, with a precision of 76.34%, recall of 65.15%, and an F1 score of 66.15%. Statistical analysis showed no significant difference between the DL model’s performance and that of the experienced dentist (*P* > 0.05), supporting its clinical reliability.

**Conclusion:**

The DL-based system successfully automated the evaluation of dental plaque from images, performing comparably to an experienced clinician. These findings underscore the potential for AI-driven plaque assessment tools to enhance digital dentistry, potentially supporting remote dental evaluations and improve oral healthcare delivery.

## Introduction

Periodontal disease is an infectious and chronic inflammatory condition [1] that poses potential risks for cardiovascular and respiratory diseases, diabetes [2], adverse pregnancy outcomes, and osteoporosis [3]. Additionally, tooth loss resulting from periodontal disease has been linked to a decline in brain function, increasing the risk of dementia [4]. As awareness of the broader health implications of oral health grows, the demand for effective dental care continues to rise.

Dental plaque, a primary cause of periodontal disease, is difficult to detect for both professionals and nonprofessionals without the use of dye-containing solutions [5]. Individual examination of each patient’s teeth is challenging as it requires considerable expertise and effort, often leading to clinician fatigue [6]. Currently, plaque detection involves the use of a detector or colored reagents, which are time-consuming and can appear unsightly during examinations [7].

In recent years, interest in research on artificial intelligence (AI) in various aspects of our society has been increasing along with the advancement of deep learning (DL). DL refers to technology that enables computers to mimic human learning and problem-solving, excelling in tasks such as classification, production, prediction, and modeling [8]. AI-driven DL systems have demonstrated superiority over previous methods in the analysis of many forms of data, such as video, audio, and text [9]. DL techniques are increasingly being applied to a wide range of complex analyses in medical imaging and diagnostic aids, owing to their consistency, scalability, and accuracy, with the goal of developing clinical decision systems to assist in lesion detection, classification, and diagnosis [10, 11]. Gulshan et al. proposed a detection algorithm for diabetic retinopathy based on retinal fundus photographs, achieving higher accuracy than ophthalmologists [12]. Similarly, Esteva et al. presented an image-based detection algorithm for skin cancer that was more accurate than the findings of dermatologists [13]. Both algorithms demonstrated the effectiveness of intelligent medical imaging that integrates AI technology.

In dentistry, AI applications are gaining traction, particularly in enhancing diagnostic efficiency and accuracy. DL systems have been used for detecting oral diseases, classifying dental lesions, and improving image quality, thereby reducing dentists’ workload and improving treatment outcomes [14, 15]. To date, most dental applications of DL have focused on analyzing X-ray radiographs to identify dental lesions or diagnostic clues [16–18]. However, radiography requires expensive equipment and involves dangers associated with radiation exposure. In contrast, DSLR images or pictures of teeth captured on mobile devices have the advantage of accessibility as they offer a cost-effective, portable alternative. While limited studies have explored DL using these imaging techniques in orthodontic patients [19], concerns persist regarding image quality due to unstructured capture techniques and unclear patient consent protocols [20].

This study aimed to develop and evaluate DL algorithms capable of automatically assessing plaque index from tooth images, providing a practical and efficient tool for dental professionals.

## Materials and methods

### Setting and participants

This study was approved by the Institutional Review Board of Nursing and Rehabilitation College of Shandong University (IRB number: 2022-R-013). The study participants comprised 70 Chinese adults (20 males and 50 females) selected from 74 volunteers. The participants were healthy individuals aged 18–55 years (mean age 25.43 years ± 7.95) with no ongoing orthodontic treatment (e.g., braces), fixed or removable prosthetic restorations, partial edentulism, extensive restorative work, significant surface staining, or severe crowding to ensure uniform dye uptake and clear plaque visualization. All participants provided written informed consent and underwent oral examination to confirm eligibility.

High-resolution dental images were captured using a Nikon D90 DSLR camera with a Nikon Micro Lens (AFS105) and GODOX ML-150 ring flash. All images were taken by the camera that was set to manual mode with a shutter speed of 1/200s, aperture of F32, and ISO 200. The white balance was set to auto, and images were taken from both frontal and lateral views with standardized positioning.

### Plaque assessment

The Quigley-Hein plaque index modified by Turesky et al., as shown in Table 1 [21], was used to evaluate plaque accumulation after the application of a plaque-disclosing agent (Plaque Check Gel BR from GC Corporation, Japan). Plaque images were captured and analyzed, and plaque scores were recorded for the buccal surfaces of 20 teeth, including the maxillary and mandibular central and lateral incisors, canines, and premolars. Plaque assessment was conducted by a single examiner with over 10 years of experience in plaque scoring. To ensure consistency, the assessment was repeated after a 2-week interval, resulting in a Fleiss kappa value of 0.876, indicating high inter-examiner reliability. Additionally, oral photographs from 14 participants were randomly selected, and plaque scores were further evaluated by a dental hygienist with 1 year of experience and a dentist with 10 years of experience.

**Table 1:** Quigley-Hein plaque index

Modified plaque scoring system of Turesky et al.	Score
**No** plaque	0
**Isolated flecks** of plaque at the cervical margin of the tooth	1
A thin continuous band of plaque **(up to 1 mm)** at the cervical margin of the tooth	2
A band of plaque wider than 1 mm covering **less than one-third** of the tooth crown	3
Plaque covering at least **one-third but less than two-thirds** of the tooth crown	4
Plaque covering **two-thirds or more** of the tooth crown	5

### Tooth data collection and labeling

Tooth images were labeled using the LabelMe program (Massachusetts Institute of Technology, Cambridge, MA, USA). Anterior teeth in frontal view images, as well as canines and first and second premolars in lateral view images, were labeled. Tooth numbers and corresponding plaque indices were recorded and used for DL model training.

To evaluate model performance, images from 59 participants (1,094 tooth images) were used for training and internal validation, with an 80:20 random split. A separate evaluation dataset consisting of 300 tooth images from 15 participants was excluded from training and used solely for performance comparison with expert evaluations.

### DL analysis for evaluating Quigley-Hein plaque index

The DL schema for evaluating the Quigley-Hein plaque index comprised three key steps: (1) Extraction of the region of interest (ROI) containing the teeth to be evaluated must be detected since the number of teeth to be evaluated is different in the frontal and buccal views. (2) Image segmentation to separate the tooth, gum, and other backgrounds in the ROI to discern each tooth. (3) Classification of segmented teeth according to the Quigley-Hein plaque index.

**Fig. 1:**
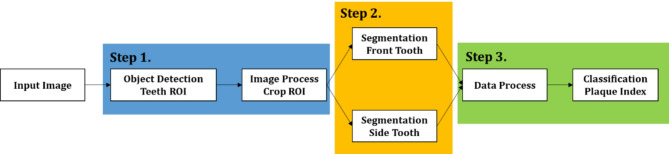
A three-step pipeline for classifying dental plaque index

The DL model was constructed in three stages, each employing a different DL structure and data type. Figure 1 shows our proposed schema for evaluating the plaque index. The first and third stages utilized both frontal and buccal tooth images to train the DL model; however, tooth segmentation in the second stage used models that separated the frontal and lateral tooth images.

**Fig. 2:**
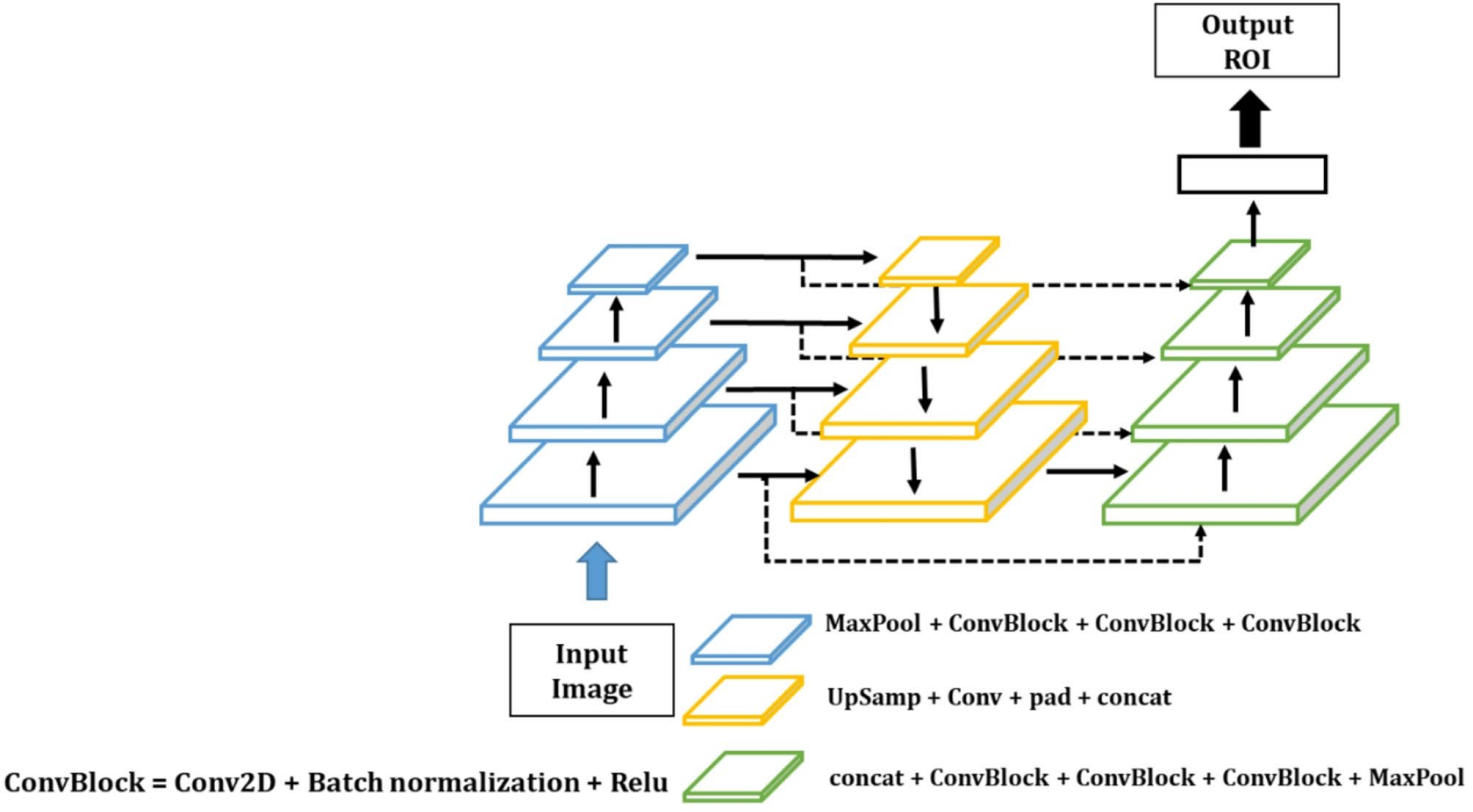
DL models of object recognition to detect tooth regions in the original tooth image

The initial stage of the process involves extracting the tooth regions. In the captured oral image, the ROI occupies a relatively small area, approximately one-quarter of the image. If a DL model were applied directly to segment the teeth from the original image, the tooth region would be too small compared to the background to receive adequate training. Consequently, an object detection model [22] is employed to identify the ROI corresponding to the tooth region, ensuring that the ratio of the tooth region to the background is accurately matched. The object detection model utilizes a convolutional neural network (CNN) [23] comprising a feature map extraction layer and a regression layer that estimates the coordinates in the image from the feature map. In our study, the Feature Pyramid Network (FPN) [24] model was employed for the extraction of the feature map, while the You Only Look Once (YOLO) [22] model was used to train the regression model, as illustrated in Fig. 2. The FPN [24] refines the feature map by establishing connections between the image pyramid structure and other components. The last regression layer detects the tooth ROI using the output structure and loss function of the YOLO model.

The training data for object recognition included images of the object from both frontal and buccal views, captured both before and after the application of a plaque-coloring dye. The model was trained using 126 frontal and 252 lateral images as training datasets, with the validation process performed using 14 frontal and 28 buccal images. The high-resolution tooth image was resized to 512 × 512 and the pixel intensities were normalized to 0–1. The output of an object detection AI model has been found to include information regarding the top, right, and bottom of the object in coordinates that have been normalized to the range of 0–1.

**Fig. 3:**
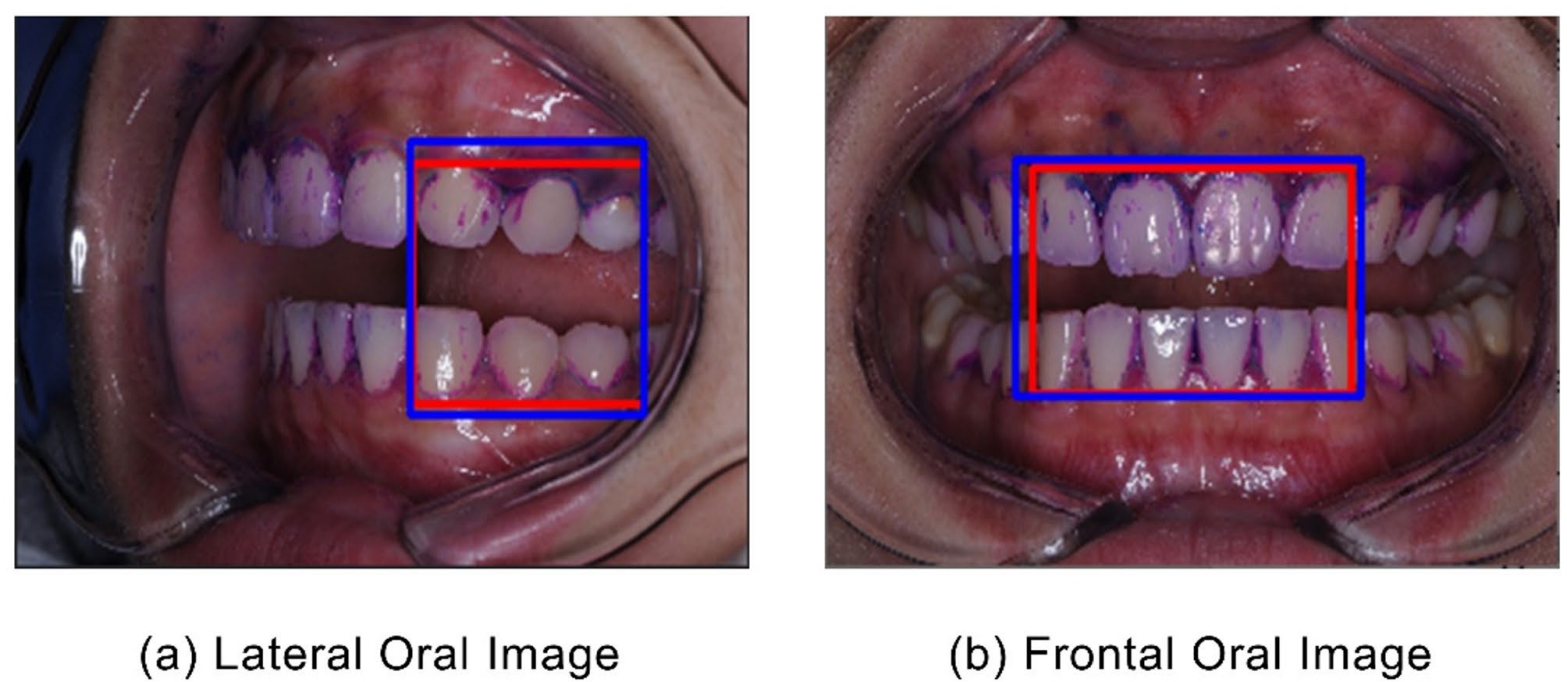
Tooth ROI results detected by object detection AI model (IOU = 86.77%, DICE = 92.78%)

The evaluation metrics employed for object recognition included Intersection over Union (IOU) [25] and Dice similarity coefficient (DICE) score [26, 27], which specifies a measurement technique that assesses the degree of overlap between the actual and predicted regions. A result approaching ‘1’ indicates a perfect match between the two regions, whereas a score of ‘0’ indicates no match. Figure 3 illustrates the results of frontal and lateral tooth ROI detection in the dental images. Red squares represent the ground truth, and blue squares represent the regions estimated by the object recognition model. The mean IOU of the validation data was 86.77%, with a DICE score of 92.78% and an overlap ratio of 86.77%. These values ensured that the tooth region to be analyzed could be reliably detected.

**Fig. 4:**
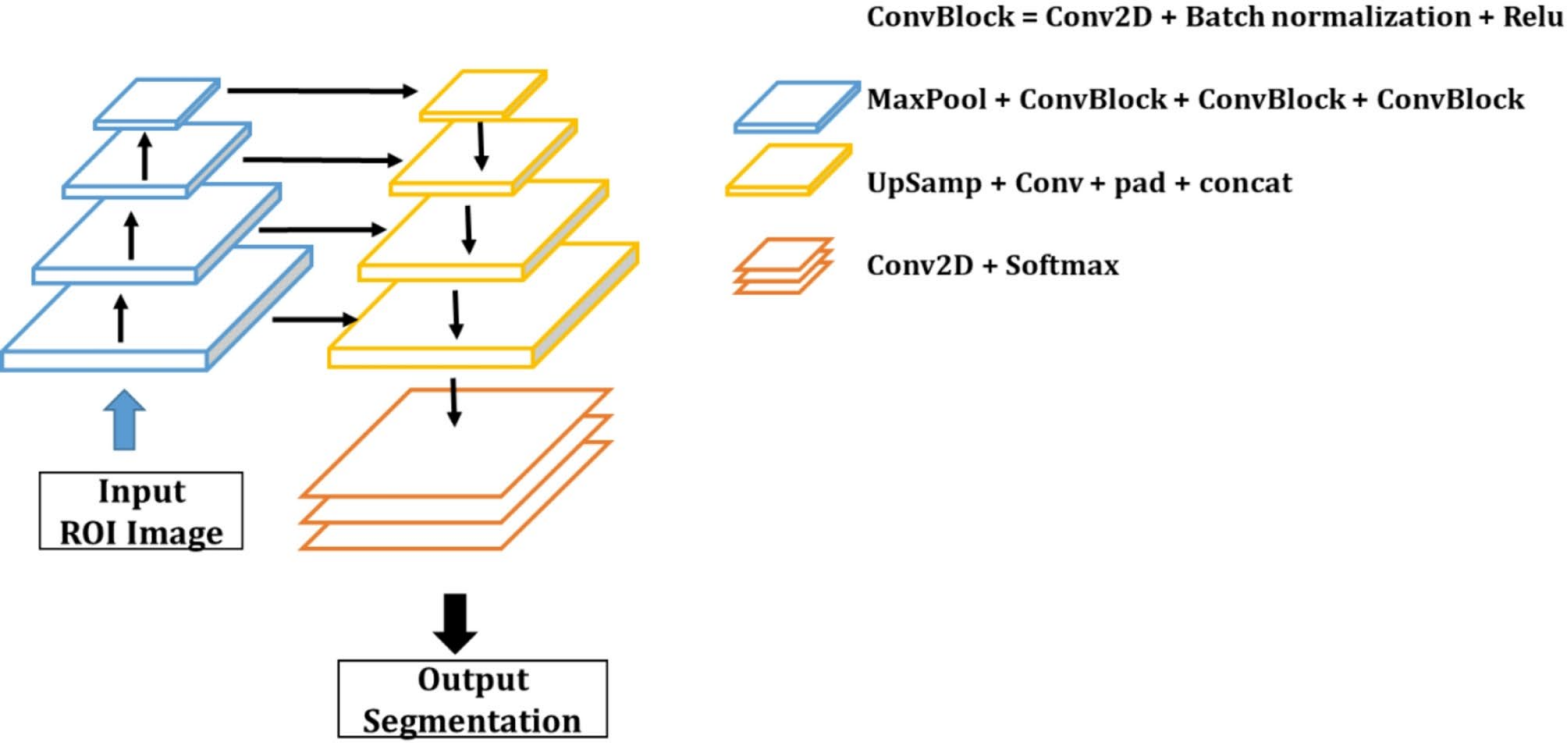
Implemented U-Net model for segmenting individual teeth

The second step involved the segmentation of the tooth image. To differentiate between individual teeth and the identified ROI, we employed the U-Net [25] DL model for tooth extraction. If the model were trained with only a binary classification of background and teeth, the boundaries of the teeth would merge, resulting in connected tooth regions. Discerning individual tooth regions requires organizing the data by separating the background, boundaries, and interiors of the teeth. Figure 4 depicts the U-Net model for separating individual teeth. It comprises four blocks of encoders, four blocks of decoders, and a final image-restoration layer.

The data used for the individual segmentation of teeth were also trained separately for frontal and buccal images. A discrepancy was observed in the segmentation of eight teeth in the frontal images and six in the lateral images. The training dataset comprised 126 frontal and 252 lateral images, while the validation dataset consisted of 14 frontal and 28 buccal images. ROIs cut from the full-size image were resized to 512 × 512 resolution and pixel normalized. The result of the AI model segmented into teeth is then processed with contour image processing to detect the outline of the tooth.

**Fig. 5:**
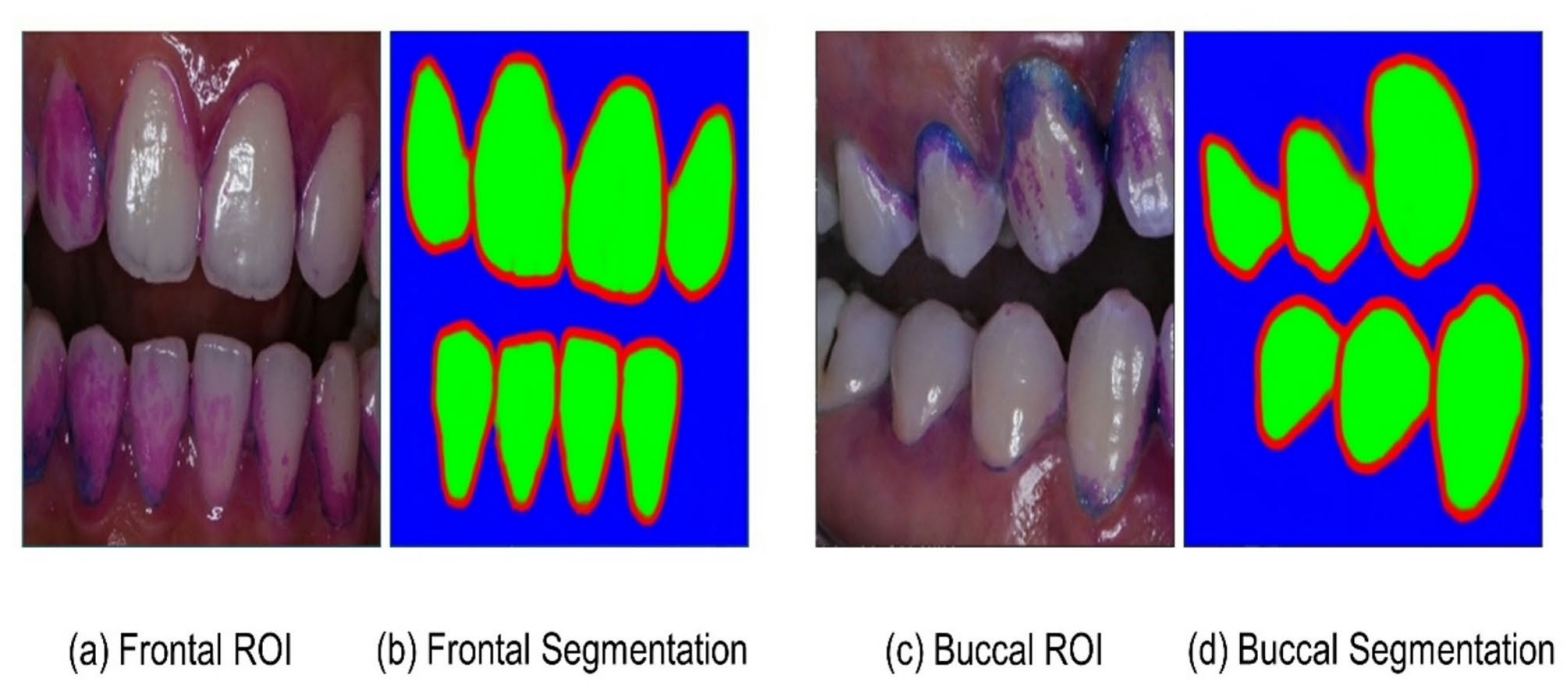
Teeth segmentation results for individual teeth from the Segmentation DL model

The DL model employed for segmentation also calculates the IOU and DICE scores, which are equivalent to the object detection metrics. As shown in Table 2, the overlap ratio of the frontal and lateral images exceeded 95% for IOU and 97% for the DICE score. Figure 5 illustrates the outcomes of tooth segmentation in the frontal and lateral regions. Figure 5(b) depicts eight correctly identified tooth regions and a single erroneous detection, which was subsequently removed during postprocessing by comparing the sizes of the regions to facilitate data organization.

**Table 2:** IOU, DICE score metrics for individual tooth segmentation models

	IOU score [%]	DICE score [%]
Frontal ROI	95.67	97.78
Buccal ROI	95.49	97.68

The final step involved categorizing the Quigley-Hein plaque index. To date, the classification problem of DL models has been addressed through various modifications to the CNN structure. Several techniques can be employed to streamline the backpropagation process in the deep layers, such as skip connections [28], depth-wise convolutions [29], dilated convolutions [30], and channel-wise weight adjustments [31]. The parameters of a configured DL model can be enhanced to determine the best-performing model depending on the depth of the CNN structure, resolution of the image, and number of filters [32].

In this study, we employed the TensorFlow framework and utilized several models, including the following: the basic CNN, residual network (ResNet) [28], The models were trained and evaluated on a workstation equipped with an NVIDIA GTX 1080 Ti GPU (11GB VRAM), AMD Ryzen 7 1700 CPU, and 32GB RAM. Atrous Spatial Pyramid Pooling (ASPP) [30], dense convolutional network (DenseNet) [33], MobileNet [29], and Squeeze-and-Excitation Network (SE Net) [31] models. We configured the learning parameters to be 10,000, 50,000, 100,000, 500,000, 1 million, and 2 million, respectively, adjusting the depth of the DL layer and the number of filters. The final layer of the configured DL model comprised the same global average pooling layer, 36 MLP layers, and MLP layers with plaque index ranges. The tooth image input to the model had a resolution of 224x224xRGB channels. During the training process, the category cross-entropy loss function and Adam optimizer were employed to update the model parameters. The total number of training epochs was 100, with learning rate changes of 0.01, 0.005, 0.001, and 0.0005 every 25 epochs. For each training epoch, the model was saved when the validation data yielded the highest accuracy.

### Statistical analysis

The sample size for this study was determined using a statistical power analysis. A significance level (α) of 0.05 and a power (1-β) of 0.8 were chosen to detect a medium effect size (Cohen’s d = 0.5). Based on these parameters, a minimum of 64 participants was required for a two-sided t-test.

To account for potential data loss and ensure reliable analysis, a total of 70 participants were recruited. This sample size was sufficient to evaluate the performance of the deep learning-based diagnostic system and facilitate comparisons with expert evaluations.

A statistical test was performed to confirm that the DL model could be matched to a human dentist. Therefore, we evaluated the difference between the results validated by the two groups on the same data using McNemar’s test [34]. As the plaque index has classes from 0 to 5, we constructed a 2x2 table with the correct and incorrect plaque index classes using the DL model and a dentist. The null hypothesis (H0) posited no significant difference between the results of the DL model and the dentist, whereas the alternative hypothesis (H1) suggested a significant difference.

## Results

### Evaluation metrics

The metrics for evaluating the machine learning classification models [35] included accuracy, precision, recall, and F1 scores. Precision is the percentage of correctly predicted labels for each class, while recall indicates the percentage of actual class data accurately identified by the model. The F1 score represents the harmonic mean of these two numbers. There are two types of accuracy: microaccuracy, which is the number of correctly predicted data divided by the total data, and macroaccuracy, which is the accuracy of each class averaged over the number of classes. Micro interpretation is common when data are uniformly distributed, but macro interpretation is more accurate when the data distribution varies significantly by class, as seen in this study. Consequently, precision, recall, and F1 scores were also calculated using macro interpretation. The plaque index classification metrics included micro accuracy, macro accuracy, precision average, recall average, and F1 score.

### Data augmentation

**Fig. 6:**
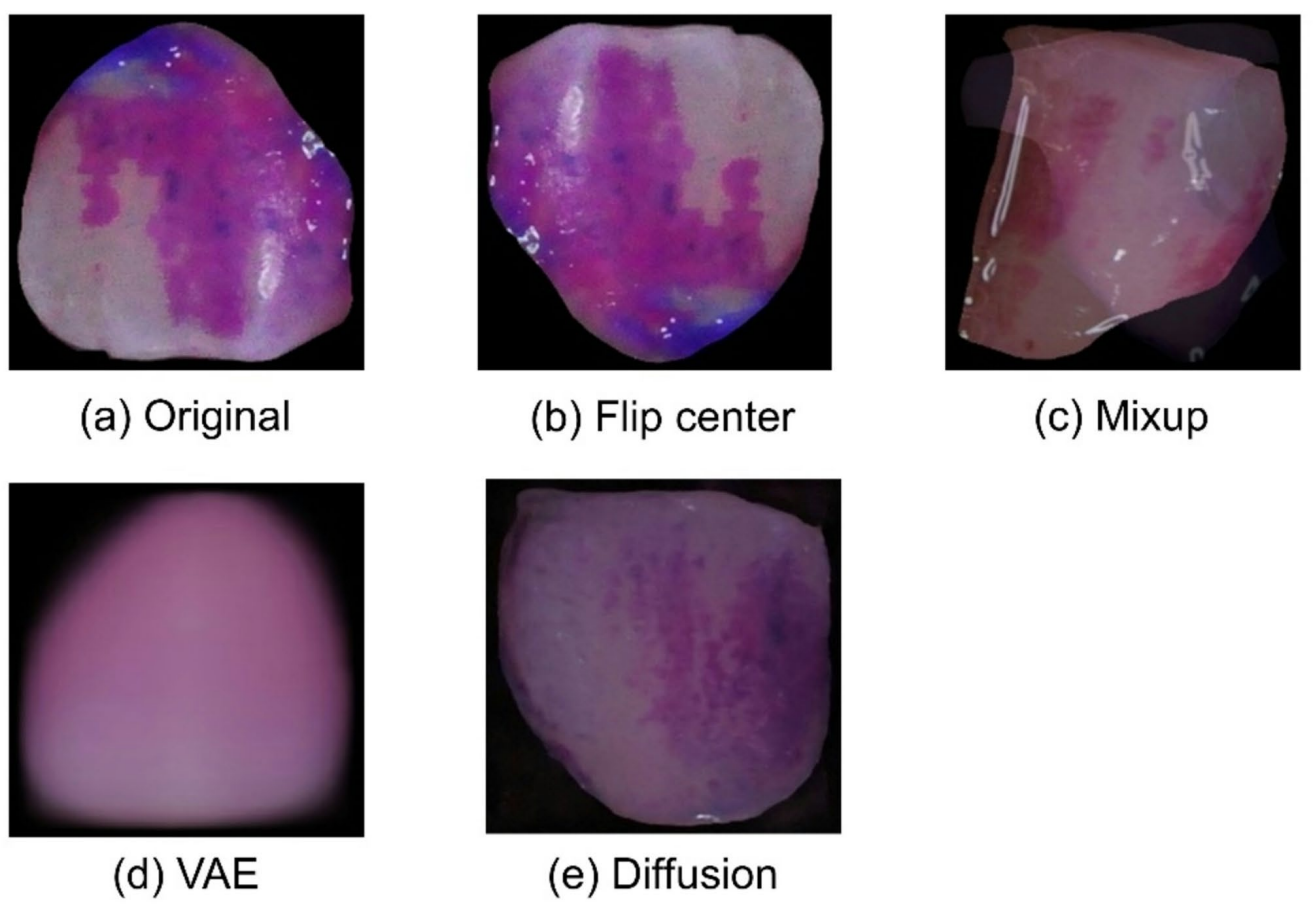
Augmented image with Quigley-Hein plaque index of 5

When teeth are individually segmented and divided according to the plaque index, there is a severe imbalance in data distribution, resulting in a significant lack of data for DL model training. To address this problem, data augmentation techniques are used, including image processing techniques and virtual data generated by generative adversarial net (GAN) models [36]. In this study, we verified the efficiency of augmentation methods for training the DL models by augmenting data. For image processing, we used flip enhancement, a method of rotating images up, down, left, and right, and the mix-up technique [37], which mixes two images of the same class and generates data using a variational autoencoder (VAE) [38] and a diffusion model [39] as generation models. The class distribution of the plaque data and the number of data generated are shown in Table 3. Figure 6 shows a sample of the augmented data for plaque index 5. The rotation augmentations were generated by reversing the left and right, up and down, up and down, left and right, and three times the original data. The mix-up and generative model data were randomly generated by focusing on the scarce data to achieve data balance. Generative model augmentation data appeared to generate images in response to the color of the plaque dye.

**Table 3:** Distribution of plaque teeth and data augmentation counts

Method	Quigley-Hein Plaque Index					
	0	1	2	3	4	5
Original	16	256	412	323	163	224
Flip	48	768	1236	969	489	672
Mixup	200	30	10	20	100	90
VAE GAN	312	263	220	243	293	285
Diffusion GAN	312	263	220	243	293	285

To evaluate the effectiveness of data augmentation, we set a CNN model with 100,000 parameters as the baseline for verification. The numbers evaluated for the validation data, starting with the original data and gradually adding augmentation methods, are listed in Table 4. The micro average indicated that the flip, mix-up, and VAE-generated models had the highest values in the data, whereas the macro average and F1 score revealed that the results using flip augmentation alone were higher. The model comparison revealed that the results of training with flip images alone were inferior to the results of training with flip, mixup, and VAE-augmented data. Therefore, we trained the original, flip, mixup, and VAE-augmented data for model and parameter validation.

**Table 4:** Assessment results for augmented data

	Micro average	Macro average	Precision	Recall	F1 score
Original	70.00	58.13	56.82	58.13	57.17
Original + Flip	71.33	66.23	74.79	66.23	67.61
Original + Flip + Mixup	73.00	66.48	66.48	66.48	66.05
Original + Flip + Mixup + Vae	73.67	65.15	76.34	65.15	66.15
Original + Flip + Mixup + Vae + Diffusion	71.00	63.14	69.36	63.14	62.95

### Comparison of plaque index assessment of DL models

Based on the outcomes of the DL models, a first-year dental hygienist and dentist with 10 years of experience validated the results of the DL classification. As shown in Table 5, the results of first-year dental hygienists’ were significantly lower than that of the 10-year dentist. Consequently, we chose the dentist’s result metric for comparison with the DL models.

**Table 5:** Plaque index assessment results from a dental hygienist and dentist

	Dental hygienist (%)	Dentist (%)
Micro Average	19.33	77.33
Macro Average	29.87	71.06
Precision	24.61	80.59
Recall	29.87	71.06
F1 score	18.37	73.20

**Fig. 7:**
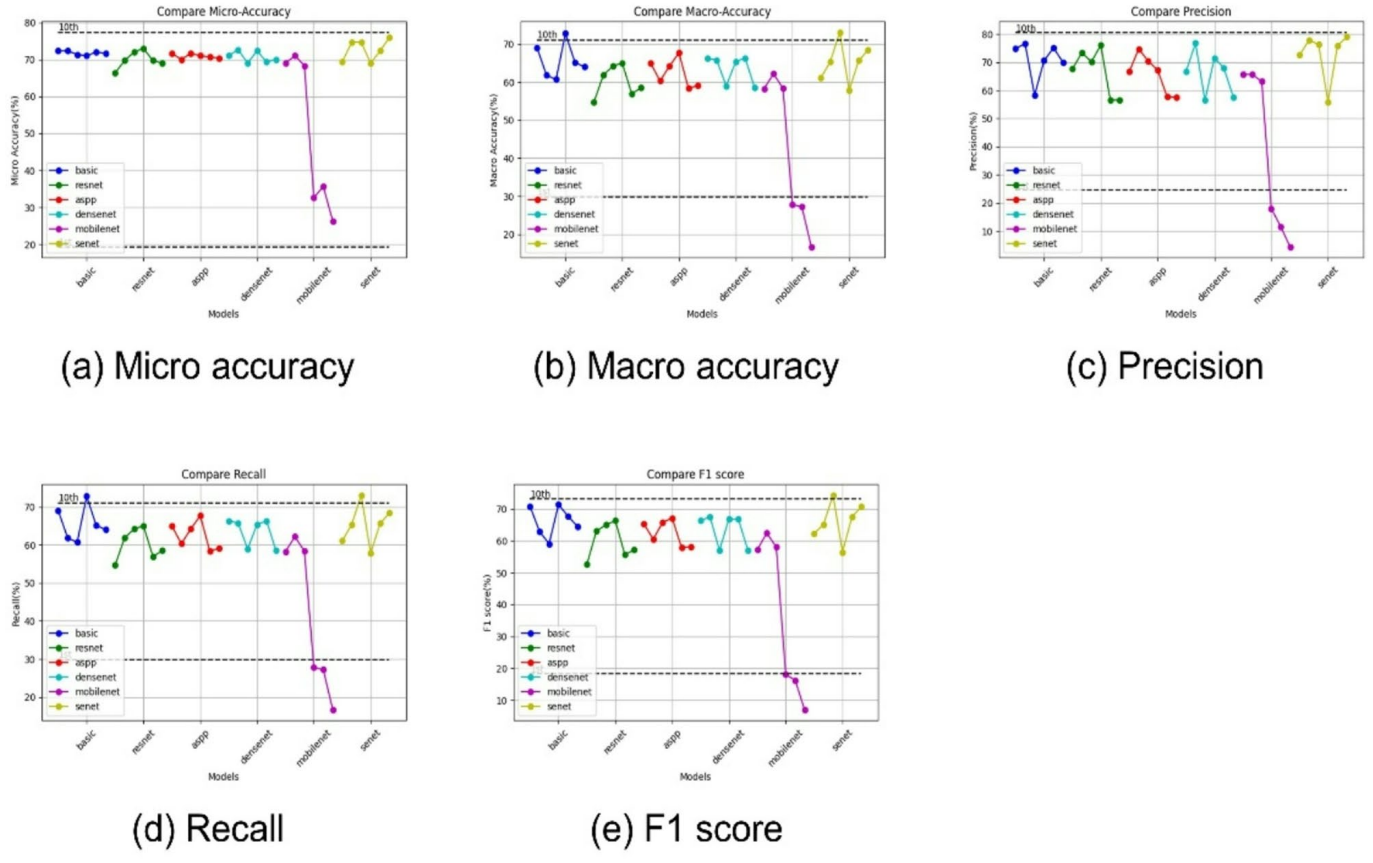
Visualize results for model structure and parameter differences

We configured each DL model with parameters of 10,000, 50,000, 100,000, 500,000, 1,000,000, and 2,000,000 by adjusting the depth of the layer, number of filters, and assigning a plaque index from 0 to 5. Table 6 shows the resulting metrics for each model, with the shaded cells indicating results that were higher than those of the dentists. Figure 7 plots the metrics in the table against those of the two evaluators. None of the models exceeded the dentist’s micro average, with MobileNet performing very poorly on the other models. However, the SE Net model with 100,000 parameters outperformed the 10-year experienced dentist in the macro average, recall, and F1 score.

**Table 6:** Results metrics for each model

Model	Index	Micro average	Macro average	Precision	Recall	F1 score
CNN	0	72.33	68.98	74.89	68.98	70.74
	1	72.33	61.78	76.54	61.78	62.88
	2	71.33	60.72	58.27	60.72	59.07
	3	71.00	72.77	70.55	72.77	71.39
	4	72.00	65.19	75.09	65.19	67.70
	5	71.67	63.99	69.94	63.99	64.52
ResNet	0	66.33	54.65	67.64	54.65	52.64
	1	69.67	61.81	73.38	61.81	63.05
	2	72.00	64.19	70.10	64.19	64.99
	3	73.00	65.02	76.09	65.02	66.31
	4	69.67	56.97	56.49	56.97	55.69
	5	69.00	58.48	56.65	58.48	57.12
ASPP	0	71.67	65.02	66.57	65.02	65.35
	1	70.00	60.31	74.72	60.31	60.53
	2	71.67	64.20	70.42	64.20	65.74
	3	71.00	67.70	67.19	67.70	67.16
	4	70.67	58.39	57.73	58.39	57.82
	5	70.33	59.09	57.48	59.09	58.16
DenseNet	0	71.00	66.33	66.67	66.33	66.30
	1	72.67	65.77	76.70	65.77	67.57
	2	69.00	58.96	56.45	58.96	56.99
	3	72.33	65.36	71.50	65.36	66.86
	4	69.33	66.35	67.95	66.35	66.77
	5	70.00	58.50	57.52	58.50	57.07
MobileNet	0	69.00	58.29	65.67	58.29	57.22
	1	71.00	62.15	65.66	62.15	62.42
	2	68.33	58.36	63.13	58.36	58.05
	3	32.67	27.85	18.08	27.85	18.05
	4	35.67	27.20	11.66	27.20	16.23
	5	26.33	16.67	4.39	16.67	6.95
SE Net	0	69.33	61.07	72.56	61.07	62.23
	1	74.67	65.37	77.85	65.37	65.19
	2	74.67	73.08	76.30	73.08	74.32
	3	69.00	57.82	55.86	57.82	56.45
	4	72.33	65.63	75.81	65.63	67.54
	5	76.00	68.46	79.06	68.46	70.67

### Diagnostic agreement between DL model and dentist

The comparison of diagnostic performance between the DL model and a human dentist using McNemar’s test is presented in Table 7.

**Table 7:** Results of the mcnemar’s table

		DL Model: SE Net	
		Correct	Incorrect
Dentist	Correct	177	55
	Incorrect	47	21

McNemar’s test, which follows a Chi-square distribution with one degree of freedom, yielded a statistical value of 0.4804, corresponding to a P-value of 0.4882. Since the P-value exceeded the significance threshold (α = 0.05), no significant statistical difference was found between the DL model and the dentist’s assessments. Therefore, we concluded that the DL model is capable of evaluating the plaque index at a level comparable to a dentist with 10 years of experience.

## Discussions

### Principal results

The DL-based model developed in this study accurately detected and classified dental plaques using the Quigley-Hein plaque index. The model achieved a micro-average accuracy of 73.67% and a macro-average accuracy of 65.15%, with a precision of 76.34% and a recall of 65.15%. This performance was comparable to that of a dentist with 10 years of experience, highlighting the model’s clinical relevance. Furthermore, the use of data augmentation techniques significantly enhanced the model’s ability to classify underrepresented plaque index categories, which is critical for maintaining high performance across diverse patient conditions.

These findings demonstrate the potential of integrating AI-driven tools into dental practice, particularly for automating plaque detection tasks that are typically time-consuming for clinicians. Automated systems can reduce the clinician’s workload, allowing for a more efficient assessment of oral hygiene status, which could ultimately improve patient care. This finding aligns with previous studies showing the utility of DL models in other medical fields where AI models have demonstrated performance at or above the level of experienced specialists [40, 41].

This performance was comparable to that of the participating dentist with 10 years of experience, based on statistical analysis, suggesting potential clinical applicability; however, generalizability remains limited and should be interpreted with caution.

### Comparison with prior work

Previous studies have predominantly focused on radiographic imaging, where AI models have been employed to detect dental lesions [42]. In contrast, our study used standard photographic images, which are easier to capture and more widely accessible, aligning with the growing interest in using AI to enhance diagnostic accuracy in noninvasive and cost-effective ways [11, 43]. Moreover, AI-based systems, such as the one developed in this study, have the potential to streamline routine dental assessments, allowing for early detection and intervention in oral hygiene management, especially in remote or underserved areas where access to dental care may be limited [44, 45].

The application of DL in dental plaque detection has gained increasing attention. Kim et al. developed a DL model that detects dental plaque from oral images using a fluorescence-based device to monitor dental plaque deposits on tooth surfaces [46]. Similarly, You et al. applied AI to assess dental biofilms in pediatric patients using intraoral cameras, demonstrating promising results in automating plaque detection and classification [7]. However, these studies relied on specific imaging techniques, such as fluorescence or intraoral cameras, which may not be practical in all clinical settings.

Previous studies on plaque detection primarily focused on assessing the degree of similarity between disclosed plaques scored on red fluorescence images and clinical plaque scores [5, 47]. In contrast, our study used a larger and more diverse dataset enhanced with data augmentation techniques to address class imbalances in plaque index categories. The DL model developed in our study achieved high accuracy, precision, and recall across multiple plaque index categories, comparable to those of a dentist with 10 years of experience.

By focusing on standard dental photography and utilizing the widely accepted Quigley-Hein plaque index, our study significantly advances the field of AI-driven plaque detection. This study aligns with the growing trend of using AI to automate routine clinical assessments, offering a scalable solution that can be easily integrated into daily dental practice and support remote dental evaluations, especially in settings with limited access to specialized equipment. Although simpler indices (e.g., Silness and Löe index or dichotomous indices) are commonly used clinically, we selected the Quigley-Hein plaque index (modified by Turesky) because its detailed 0–5 scale provides richer visual data for training the deep learning model. This detailed classification enables the algorithm to capture subtle variations in plaque distribution, enhancing accuracy. Importantly, this detailed scale does not limit clinical use, as the model could easily be adapted to simpler indices or binary classifications according to clinical preferences and practical requirements.

We evaluated the plaque detection performance of the DL models, with DL performance metrics assessed by separating the training and validation data based on their data distribution. However, owing to the scarcity of plaque index scores of 0 in the collected dataset, it was challenging to assert that the DL models were adequately trained. A key limitation in the studies conducted by Chen et al. [48] stems from a biased selection of the training dataset, in which the validation data can affect the plaque index decisions of DL models owing to the random separation of training or validation datasets among the entire collection of raw data. Additionally, their study [48] lacked clinical evaluations of patient’s plaque conditions by real dentists. Consequently, there was no comparison between the DL model’s performance and clinical decisions. These findings underscore the need to critically validate the applicability of DL models through clinical comparisons with dentists’ evaluations.

When the training and validation data were split between subjects for clinical validation, there was a significant lack of data on low plaque index scores. To address this problem, we employed general image rotation for additional training; however, we confirmed that this method was not effective for insufficient data. A slight improvement was achieved in the performance by adding mixed image processing and generative models. Additionally, commonly used classification models grow to more than 1 million parameters when layered at a small depth. Therefore, when using insufficient data and heavy DL models, overfitting often occurs without properly learning the features of plaque tooth images. Consequently, we trained and validated models with different structures ranging from 10,000 to 2 million parameters and found that most DL classification models with 50,000 to 100,000 parameters performed well. Notably, the MobileNet demonstrated a sharp decline in performance as the number of parameters increased. In a situation with limited data, studies using DL models can only be considered reliable if the DL structure and size are verified simultaneously.

As earlier mentioned, Chen et al. [48] evaluated DL models using randomly extracted datasets without considering clinical evaluation. Their study lacked evidence that the performance indicators of their DL models were meaningful in terms of clinical decisions. Therefore, our study aimed to address the dental-decisive verification of the DL model performance over dental hygienists and dentists and highlight its clinical significance. We compared the performance of the best DL model against that of a dentist with 10 years of experience. Our best DL model did not outperform the dentists in all metrics, and the difference test showed no significant differences. However, the DL model was on par with a dentist with 10 years of experience and outperformed a dental hygienist with 1 year of experience, proving that the DL model can be used as an adjunct indicator of plaque assessment even for non-experts.

The validation process of our DL model involved only one experienced dentist and one dental hygienist, which limits the robustness of inter-rater reliability. Future studies should include multiple experienced evaluators to strengthen ground truth accuracy and enhance the clinical validity of the automated assessment. Our dataset consisted of participants recruited from a specific geographic area, potentially limiting demographic diversity.

### Limitations and future work

This study has some limitations. First, the sample size of 70 participants may have limited the generalizability of the findings. Although these findings are promising, a larger and more diverse population is necessary to further validate the model’s performance across different demographic and clinical backgrounds. Second, we restricted imaging to the buccal surfaces of incisors, canines, and premolars and applied strict inclusion criteria that excluded participants with fixed or removable prosthetic restorations, orthodontic appliances (e.g., braces), or partial edentulism in order to ensure uniform dye uptake and clear plaque visualization. This focus provides high-quality input for initial model development but limits applicability to patients with restorations, braces, edentulous areas, or posterior/lingual surfaces. Third, the use of high-resolution DSLR cameras to capture images under controlled conditions ensures high-quality images for model training and evaluation, it may not reflect the variability encountered in real-world clinical settings, where mobile devices or intraoral cameras are more commonly used. This could potentially affect the model’s accuracy when applied outside controlled environments. Future studies should assess the model’s performance using different imaging devices under varied conditions to ensure robustness. Fourth, manual labeling of images for training the model introduces potential human error. Although efforts were made to maintain consistency through repeated assessments and to achieve a high inter-examiner reliability score (Fleiss’s kappa of 0.876), there remains the potential for bias in the initial plaque scoring. Especially, our study did not include calibration or consensus scoring among multiple expert evaluators, relying instead on one experienced examiner. This introduces potential subjectivity and variability, particularly with the multi-level plaque index used. The model’s performance was compared to only one dentist with 10 years of experience, which limits generalizability and calls for further validation with multiple experts. Future research should incorporate calibration among multiple evaluators to improve reliability and generalizability. Overall, Future work will expand the inclusion criteria to include participants with prostheses, orthodontic appliances, or partial edentulism; incorporate full-mouth imaging; and validate performance on patient-captured images in real-world contexts that support remote dental evaluations.

## Conclusion

Manually assessing the plaque index can be a time-consuming and labor-intensive task, even for experienced dentists. This study highlights that automatic plaque assessment using DL is clinically effective for identifying meaningful diagnostic features. We demonstrated the feasibility of automated plaque detection by implementing a DL model to extract tooth regions and assess the plaque index, providing a valuable tool for efficient and objective plaque detection in clinical practice.

## Data Availability

The data that support the findings of this study are available from the corresponding author upon reasonable request.

## Abbreviations

AI: Artificial intelligence
DL: Deep learning
ROI: Region of interest
CNN: Convolutional neural network
YOLO: You Only Look Once
FPN: Feature Pyramid Networks
IOU: Intersection over union
DICE: Dice similarity coefficient
VAE: Variation Auto Encoder
ResNet: Residual nets
ASPP: Atrous Spatial Pyramid Pooling
DenseNet: Dense Convolutional Network
SE Net: Squeeze-and-Excitation Networks
